# The Forgotten Fruit: A Case for Consuming Avocado Within the Traditional Mediterranean Diet

**DOI:** 10.3389/fnut.2020.00078

**Published:** 2020-05-29

**Authors:** Nikki A. Ford, Ann G. Liu

**Affiliations:** ^1^Avocado Nutrition Center, Mission Viejo, CA, United States; ^2^Independent Researcher, Valencia, CA, United States

**Keywords:** avocado, *Persea americana*, Mediterranean, dietary pattern, plant-based, oleic acid

## Abstract

The Mediterranean diet is rich in fruits and vegetables and includes an abundant intake of oleic-acid-rich olive oil. People who adhere to a Mediterranean diet have reduced risk for numerous chronic diseases. As obesity rates rise globally, people who choose to follow a traditional Mediterranean diet and/or make improvements in food choices may reduce their risk of metabolic dysfunction and disease. Incorporating non-traditional fruits and vegetables into the Mediterranean diet could provide greater flexibility in suitable food choices for people who struggle to adhere to recommended healthy dietary patterns, and it could also provide greater adaptability for people living outside of the Mediterranean region who are interested in adopting the diet. The avocado fruit thrives in a Mediterranean climate, is produced in the region, and is rich in oleic acid and fiber, yet avocados are not commonly consumed within the traditional Mediterranean diet. Based on the existing research studies on the health benefits of avocado consumption and the continued investigation into the nutritional attributes of the avocado, a case can be made for including avocados as part of the Mediterranean dietary pattern.

## Introduction

Across the world, countries are experiencing major shifts in their food systems and diets that affect the nutritional status and health of their inhabitants. In the Mediterranean region, the traditional dietary pattern is mainly plant-based with (1) high consumption of vegetables, fruits, legumes, unrefined grains, nuts, and olive oil, (2) moderate intake of fish, wine, and fermented dairy products such as cheese and yogurt, and (3) low intake of meat and processed foods (1–3). Higher levels of adherence to the Mediterranean diet are associated with reduced risk of inadequate nutrient intake, indicating that the diet provides a variety of essential nutrients (4). However, for decades many Mediterranean countries have been drifting away from this traditional Mediterranean dietary pattern with increased intake of animal products and ultra-processed foods and decreased consumption of plant-based foods (5, 6).

This change in diet has health implications for the Mediterranean population because adherence to the Mediterranean diet is associated with numerous health benefits, including reduced risk of several major chronic diseases. There is consistent evidence that better adherence to the Mediterranean diet is associated with clinically meaningful reductions in rates of coronary heart disease, ischemic stroke, and total cardiovascular disease (CVD) (7). A 2-point increase in adherence to the Mediterranean diet is associated with an 8–9% reduction in overall mortality and a 10% reduction in risk of CVD (8, 9). Higher adherence to a Mediterranean diet is also associated with a 19–23% reduction in the risk of developing diabetes (10, 11) and a reduced incidence of cancers (8, 9, 12). Adherence to a Mediterranean diet may also reduce the risk of neurodegenerative diseases such as Parkinson's disease and Alzheimer's disease (8, 13).

Because the Mediterranean diet describes a general dietary pattern of a large and diverse region, which includes 22 countries, there is no single version of the Mediterranean diet. It varies with geography and historical time, and it is adapted by each specific geographic area and population (14). As the food systems and availability in a region change, dietary patterns may change to reflect this. The availability and consumption of non-traditional foods have been increasing in the Mediterranean region, and one potential way of shifting the dietary pattern back to a more healthful nutrient profile is to encourage intake of fruits and vegetables regardless of whether they are traditionally consumed in the Mediterranean region. Incorporating non-traditional foods such as avocados into the Mediterranean dietary pattern could provide greater flexibility in suitable food choices, and provide greater adaptability for populations outside of the Mediterranean region.

## Nutritional Attributes of Avocados

Avocados (*Persea americana*) are nutrient-dense and botanically considered a fruit, but they are not traditionally included in the Mediterranean diet. Avocados may be pear-shaped, egg-shaped, or spherical, and they consist of a single large seed surrounded by a creamy, smooth textured, edible fruit and are covered by a thick, bumpy skin that turns purplish black when ripe. The Mediterranean diet traditionally includes high consumption of fruits and vegetables, and the World Health Organization (WHO) recommends eating at least 400 g, or five portions, of fruit and vegetables per day to reduce the risk of non-communicable diseases and to ensure adequate intake of dietary fiber (15). A serving of avocado (50 g or ~1/3 of a medium-sized fruit) contains 3.4 g dietary fiber (11% DV), 44.5 μg folate (10% DV), 0.73 mg pantothenic acid (15% DV), 85 μg copper (10% DV), and 10.5 μg vitamin K (10% DV) (16). Avocados have a low energy density of 1.6 kcals/g or 80 kcals/serving. One serving of avocado contains 5 g monounsaturated fatty acid (MUFA) and 1 g polyunsaturated fatty acid (PUFA), with the predominant fatty acid being oleic acid at 4.53 g/serving (16). Avocados also contain numerous bioactive phytonutrients that may impart health benefits (17–21).

Table 1 shows the nutrient composition of avocados compared to other commonly consumed foods which are part of the Mediterranean diet. Both avocados and olive oil are rich in oleic acid, and they have a similar ratio of unsaturated to saturated fat (6:1) while avocados simultaneously provide other important nutrients. Like avocados, nuts are also a source of fiber, potassium, and folate, but they have a high energy density with walnuts providing 185 kcal/serving which is two times higher than the amount of energy provided by a serving of avocado.

**Table 1:** Nutrient composition of avocados compared to commonly consumed foods in the Mediterranean diet.

**Food (NLEA serving)**	**Energy (kcal)**	**Fat (g) [unsaturated: saturated ratio]**	**Fiber (g)**	**Sugar (g)**	**Potassium (mg)**	**Folate (mcg)**
Avocado^a^ (50 g)	80	8 [6:1]	3	0	254	45
Olive oil^b^ (13.5 g/1 tbsp)	119	14 [6:1]	0	0	0	0
Walnuts^c^ (28 g/1 oz)	185	19 [8:1]	2	3	125	98
Apples^d^ (242 g)	126	0	6	25	259	7
Tomatoes^e^ (148 g)	27	0	2	4	351	22

Nutrient data obtained from U.S. Department of Agriculture, Agricultural Research Service. FoodData Central, 2019. https://fdc.nal.usda.gov.

a Avocados, raw, California.

b Oil, olive, salad, or cooking.

c Nuts, walnuts, English.

d Apples, raw, with skin.

e Tomatoes, red, ripe, raw, year round average. Apples are the most popular fruit choice purchase in the EU, and tomatoes are the most popular vegetable.

*NLEA, Nutrition Labeling and Education Act*.

## Health Benefits of Avocados

Population data demonstrate the health benefits of consuming more fruits and vegetables. A meta-analysis of 95 unique cohort studies found inverse associations between high intakes of fruits and vegetables and risk of CVD, certain cancers, and all-cause mortality (22). Fruit intake has also been shown to be associated with reductions in body weight, waist circumference, and risk for obesity (23). Evidence from randomized controlled trials suggests that increasing the consumption of fruits and vegetables has favorable effects on CVD biomarkers such as blood pressure and lipids (24). Avocados and olives are unique among fruits in that they contain high levels of fat, specifically MUFA. Numerous organizations, including the WHO, American Heart Association, and 2015–2020 Dietary Guidelines for Americans, recommend replacing saturated and trans fats with MUFA and PUFA to reduce the risk of CVD (25–27). Substituting saturated fats with unsaturated fats is also associated with a significant reduction in total mortality (28). Previous clinical studies provide evidence suggesting that eating avocados may yield health benefits (29), and here we provide an update on recent research in the area.

### Nutrient Status

Avocado consumption is associated with better diet quality and increased intake of important nutrients. Analysis of data from the National Health and Nutrition Examination Survey (NHANES) revealed that among U.S. adults, avocado consumption was associated with better diet quality, including significantly higher intake of fruits, and vegetables, and a lower intake of added sugars (30). Avocado consumers also had significantly higher intakes of dietary fiber, vitamins E and K, magnesium, and potassium. The average avocado intake by consumers was 70 g/day, which is equivalent to about half of a fruit.

Avocados can also increase the absorption of lipid-soluble bioactive phytonutrients such as carotenoids. Adding avocado to salsa enhances the absorption of lycopene and β-carotene, while adding avocado to a salad increases absorption of α-carotene, β-carotene, and lutein (31). Adding avocado to a meal increased the absorption of β-carotene from both tomato sauce and raw carrots, and increased the efficiency of bioconversion (5–13 fold increase) of β-carotene to vitamin A (32). Thi suggests that consuming a lipid-rich food with vegetables high in β-carotene may be especially important for people with low vitamin A status (32).

Avocado consumption enhances the absorption of the carotenoids lutein and zeaxanthin, which accumulate in the eyes and brain. Lutein and zeaxanthin accumulate in the macula of the retina and retinal lutein concentrations (macular pigment density) are correlated to brain lutein concentrations (33). In a randomized controlled trial, eating one avocado per day for 6 months increased serum lutein by 0.93 nmol/L and increased macular pigment density by 0.101 OD compared with the control group which consumed potatoes or chickpeas (34). Interestingly, previous work from the same authors, found that lutein supplementation of 12 mg/day for 4 months increased serum lutein by 0.22 nmol/L and macular pigment density by 0.041 OD (35). Therefore, even though avocados contain a small fraction of the amount of lutein found in the supplement, they were far more effective at increasing serum lutein and macular pigment density. This is likely because avocados contain MUFA, which enhance the absorption of carotenoids, and previous data has shown that consuming avocados modulates lipoproteins that are responsible for carotenoid transport (36). Eating avocados may also contribute to brain and eye health since macular pigment density is related to cognitive function in adults (33). Specifically, in the 6-months intervention trial participants consumed ~500 μg of lutein daily from avocado, which led to increases in serum lutein and macular pigment density (34). In the avocado group, these changes in macular pigment density correlated with improvements in spatial working memory and efficiency in approaching a problem. These results align with previous research showing that higher lutein status is related to better cognitive performance (33).

### Cardiometabolic Health

CVD is the leading cause of morbidity and mortality worldwide, but the risk of CVD can be meaningfully reduced by consuming a healthy diet that emphasizes fruits, vegetables, legumes, nuts, whole grains, and fish (26). Eating avocado as part of a healthy diet has been shown to modify some cardiometabolic risk markers such as lipid profile (29). In an analysis of NHANES data, avocado consumers had significantly higher high-density lipoprotein cholesterol (HDL-C) levels and a 50% lower odds ratio for metabolic syndrome (30). Potentially beneficial effects of avocados on lipid profile have also been seen in meta-analyses. Mahmassani et al. conducted a meta-analysis of 18 studies examining avocado consumption and cardiovascular risk factors, and they found that avocado intake was associated with increased HDL-C (37). A previous meta-analysis by Peou et al. included 10 studies assessing avocado intake and plasma lipoproteins, and found that avocado intake was associated with reduced total cholesterol, low-density lipoprotein cholesterol (LDL-C), and triglycerides (38). The difference in findings may be due to variations in data analyses. Mahmassani et al. evaluated the net change in serum lipids (change in the avocado arm—change in the control arm) whereas Peou et al. only compared changes in the avocado arms without accounting for changes in the control arms. Though the methods and findings differ somewhat, these studies indicate that avocado consumption likely alters the lipid profile.

Randomized controlled trials have also demonstrated that eating avocado can improve lipid profiles. In a randomized, crossover, controlled feeding study, consuming a moderate-fat diet containing one avocado a day for 5 weeks reduced LDL-C and non-HDL-C more than a moderate-fat diet or a low-fat diet (36). Moreover, only the avocado-containing diet significantly reduced the number of small dense LDL-C particles, oxidized LDL-C, and the ratio of LDL-C/HDL-C (39). Another randomized, crossover, controlled feeding study found that consuming a whole avocado (136 g), as part of a breakfast meal, lowered concentrations of triglyceride-rich lipoproteins, and increased larger HDL-C particles, compared to an energy-matched, higher-carbohydrate meal (40). Eating either a whole or a half avocado as part of breakfast also significantly reduced postprandial glycemic and insulinemic responses compared to a control meal. Similarly, in a randomized, crossover study, Wien et al. compared a control meal to a lunch that included avocado (isocaloric) or avocado added to the lunch (+112 kcal) on insulin and glucose levels (41). The insulin area under the curve (AUC) was lowest when avocado was exchanged for other components of the meal (isocaloric). These studies suggest that avocado consumption may augment cardiometabolic risk markers.

### Weight Management

Obesity rates have been increasing worldwide for several decades, and a healthy dietary pattern is an important component of weight loss and weight management. In the U.S. population, avocado consumers have lower body weight, body mass index (BMI), and waist circumference compared with non-consumers (30). Avocado consumers are also less likely to be overweight or obese and less likely to have an elevated waist circumference compared with non-consumers. Specifically, analysis of the 2001–2012 NHANES dataset reported avocado consumers were 33% less likely to be overweight or obese and 32% less likely to have an elevated waist circumference compared to non-consumers (42). Moreover, on average, avocado consumers weighed 3.4 kg less, had a mean BMI of 1 unit less, and had a waist circumference 3.0 cm smaller compared to non-consumers. A longitudinal study of ~55,400 7th-day Adventists in the U.S. in Canada found that avocado consumers gained significantly less weight over time (4–11 years follow-up) than non-consumers (43).

One possible explanation for these findings is that including avocados may increase satiety and reduced hunger when included in meals. A randomized, controlled clinical trial demonstrated that participants could eat one whole avocado daily without derailing their weight loss efforts (44). Study participants who ate an avocado daily as part of a hypocaloric diet maintained a similar feeling of satiety throughout the study compared to a reduction in satiety reported by participants on the control diet. Wien et al. found participants reported increased meal satisfaction and reduced desire to eat following a lunch that added half an avocado compared to eating the meal with no avocado (41). In the study by Zhu et al. participants reported increased satisfaction associated with eating a breakfast meal which included a half or a whole fresh avocado, compared to an isocaloric, high-carbohydrate control meal (45). Hunger was also significantly suppressed by the whole avocado-containing meal. These studies only examined subjective measures of appetite, and further research would be needed to determine if there are corresponding changes in caloric intake. The body of evidence thus far suggests that avocados could be a good addition to the Mediterranean diet pattern as there is evidence suggestive of health benefits and no evidence of harm.

## Observational Datasets to Assess the Role of Avocado, as Part of a Mediterranean Diet, on Health

There are several challenges of using existing observational data to assess the impact of an avocado-containing dietary pattern on health. One challenge is that avocado intake is poorly captured in existing datasets. Countries that historically have consumed the most avocados (Mexico and other Latin American countries) do not have adequately funded federal programs to capture the information. In the U.S., the NHANES dataset uses two 24-h dietary recalls, which fail to adequately capture the intake of foods that are sporadically consumed, like avocados. Other large datasets that capture a range of avocado intakes, such as the Adventist Health Study-2 or the Hispanic Community Health Study/Study of Latinos, do not represent the general demographics of the U.S. population. Another challenge is potential confounding with other commonly co-consumed foods. Avocados are most commonly consumed as guacamole, which may be co-consumed with a dipper such as tortilla chips or vegetable sticks. Avocados are also frequently used in salads or as an add-on to sandwiches. The co-consumption of avocados with other foods and changes in the preferred methods of consumption over time may make data analysis challenging. A final challenge is that a biomarker of intake does not exist for avocados, so there is currently no objective way to validate self-reported consumption; however, given that avocado consumption is associated with better diet quality and avocados also have potential health benefits, adding avocados to clinical food assessments, and diet scores, may improve disease/mortality predictions at the population level.

## Model of Avocado Exchanges in a Mediterranean Dietary Pattern

To date, no observational or intervention study has investigated the role of avocados within a Mediterranean dietary pattern. A simple dietary food pattern was developed (Table 2) which represents a typical 3-days Mediterranean diet with or without avocado. In this isocaloric model, avocado was exchanged for fruit, vegetable, oil, nuts, dairy, and legumes in reasonable culinary swaps. In this model, the avocado-inclusive Mediterranean diet provided slightly less sugar (−7 g/d), sodium (−225 g/d), vitamin D (3 IU/d), and calcium (−132 mg/d) with an increase in fiber (+10 g/d), plant sterols (+114 mg beta-sitosterol/d), folate (+124 mg/d), and potassium (+552 mg/d). Nutrient intake would differ in other models which include varying amounts of avocado. Also, nutrient and food group intake would differ with other reasonable culinary exchanges in the food pattern. As an example, a diced avocado relish can be used on top of chicken or fish, or avocados can easily be added to salads and soups. Avocados can replace some spreads and baking ingredients to increase nutrient density and improve the ratio of unsaturated to saturated fatty acids.

**Table 2:** Comparison of a 3-days typical Mediterranean dietary pattern compared to a typical Mediterranean dietary pattern that includes one avocado a day.

**Meal**	**Day 1**	**Day 2**	**Day 3**
**Breakfast**	Avocado, raw (50 g)^a^	Yogurt, Greek, plain, non-fat (6 oz)	Whole wheat bread, toasted (1 slice)
	Egg, chicken, poached (1 large)	Walnut, black, dried (1/2 oz)	Orange and apricot juice (4 fl oz)
	Whole wheat bread, toasted (1 slice)	Grapefruit juice, pink, raw (1/2 cup)	Avocado, raw (50 g)^g^
		Blueberry, raw (1/2 cup)	
**Snack**	Apple with skin, raw (1 medium)	Apple with skin, raw (1 medium)	Banana, raw (1 small)
	Pistachio nut, dry roasted, no salt (1 oz)	Almond butter, no salt (1 Tbsp)	
**Lunch**	Capers (1 Tbsp)	Vinegar, balsamic (1 Tbsp)	Vinegar, balsamic (1 Tbsp)
	Feta cheese, crumbled (2 Tbsp)	Tuna, blufin, fresh, cooked (2 oz)	Red pepper, sweet, sauteed, chopped (1/2 cup)
	Lentil, boiled, no added salt (1 cup)	Tomato, red, ripe, raw (1/2 cup)	Pita bread, whole wheat (1 large)
	Pear, green anjou, raw (1 small)	Orange, all varieties, raw (1 cup)	Olive oil (1/2 Tbsp)^*^
	Olive oil (2 Tbsp)	Olive oil (1 Tbsp)	Mushroom, portabella, grilled (3/4 cup)
	Red pepper, sweet (1/2 cup)	Lettuce, cos or romaine, raw (2 cup)	french fries, sweet potato (2 oz)
	Tomato, sun-dried, packed in oil (1/4 cup)	Dinner roll, whole wheat (1 medium)	Avocado, raw (50 g)^h^
	Vinegar, balsamic (1 Tbsp)	Chickpea (Garbanzo bean), low sodium (3 Tbsp)	
		Carrot, raw, grated (1/2 cup)	
		Avocado, raw (50 g)^d^	
**Snack**	Avocado, raw (50 g)^b^	Whole wheat cracker, low salt (1 oz)	Snacks, pita chips, salted (1 oz)
	Carrot, raw, sliced (1 cup)	Avocado, raw (50 g)^e^	Avocado, raw (50 g)^i^
	Lemon juice, raw (1 tsp)		
**Dinner**	Avocado, raw (50 g)^c^	Zucchini with skin, raw, sliced (1 cup)	Vinegar, balsamic (1 Tbsp)
	Kale, boiled, no salt, chopped (1 cup)	Red pepper, sweet (1/2 cup)	Tomato, red, ripe, raw (1/2 cup)
	Olive oil (1 Tbsp)	Pesto with basil (1 Tbsp)	Spinach, raw (2 cup)
	Quinoa, cooked (3/4 cup)	Olive (6 small)	Salmon, Atlantic, farmed, cooked (6 oz)
	Salmon, Atlantic, wild, cooked (3 oz)	Olive oil (1/2 Tbsp)^*^	Pistachio nut, dry roasted, no salt (1 oz)
	Tomato, red, ripe, cooked (1/2 cup)	Mushroom, raw (1/2 cup)	Olive oil (1/2 Tbsp)
		Chicken breast, skinless, boneless, meat only, grilled (4 oz)	Bulgur wheat (1/3 cup)
		Brown rice, medium grain, cooked (6 oz)	
		Avocado, raw (50 g)^f^	
**Nutrients and food groups**	**Typical Mediterranean diet: 3 days mean** **±** **SD**	**Typical Mediterranean diet with Avocado Swaps: 3 days mean** **±** **SD**	**Difference in intake**
Energy (kcal)	1,972 ± 57	1,960 ± 70	−13
Energy (KJ)	8,250 ± 237	8,199 ± 290	−51
% Cal Protein	19 ± 2	19 ± 3	0
% Cal Carb	35 ± 2	34 ± 2	−1
% Cal Fat	46 ± 2	47 ± 4	1
Sat Fat (g)	18 ± 1	15 ± 2	−3
Mono Fat (g)	49 ± 7	51 ± 8	2
Poly Fat (g)	20 ± 2	20 ± 4	0
Sugar (g)	63 ± 21	55 ± 34	−7
Fiber (g)	37 ± 10	47 ± 6	10
Cholesterol (mg)	193 ± 65	176 ± 79	−17
β-sitosterol (mg)	52 ± 13	166 ± 13	114
Folate (mcg)	442 ± 153	567 ± 127	124
Vitamin D (IU)	23 ± 19	20 ± 21	−3
Calcium (mg)	602 ± 144	470 ± 178	−132
Potassium (mg)	3,748 ± 526	4,300 ± 259	552
Sodium (mg)	1,937 ± 682	1,713 ± 240	−225
Iron (mg)	16 ± 5	16 ± 4	0

Analyzed by Heidi Diller, RDN in Orange, CA on March 11, 2020. Software: Nutribase 19 Pro Edition V19.2. Utilizing only food values from the USDA National Nutrient Database.

* olive oil adjusted for energy needs: Day 2 Dinner (1.5 T reduced to 1/2 T); Day 3 Lunch (1T reduced to 1/2 T).

a 1/3 of a medium avocado (50 g) replaced honeydew melon, raw, dice (1 cup).

b 1/3 of a medium avocado (50 g) replaced olive, small-extra large (3 oz).

c 1/3 of a medium avocado (50 g) replaced pinenut, pinyon, dried (1/2 oz).

d 1/3 of a medium avocado (50 g) replaced olive oil (1 Tbsp).

e 1/3 of a medium avocado (50 g) replaced grape, American-type, raw (1/2 cup).

f 1/3 of a medium avocado (50 g) replaced feta cheese, crumbled (1 oz).

g 1/3 of a medium avocado (50 g) replaced peanut butter, smooth (1 Tbsp).

h 1/3 of a medium avocado (50 g) replaced swiss cheese (1 oz).

i 1/3 of a medium avocado (50 g) replaced hummus, raw (1/4 cup).

## Production and Consumption of Avocados

While avocados are a healthy and nutritious fruit, they are also well-suited for incorporation into the Mediterranean diet because production and consumption of avocados are increasing in the region. Although production of avocados in the Mediterranean region has historically been low, avocado trees thrive in a Mediterranean climate. Spain has the highest production in the Mediterranean region with ~67,000 tons produced in 2017 compared to 36,000 tons produced in 2014 (46–48).

Avocado consumption is increasing in many countries around the world with approximately half of all avocados consumed in the United States, a third consumed in the European Union, and 20% consumed in other world markets (47). In areas where avocado intake is increasing rapidly, such as the Mediterranean, fruit needs to be imported from outside the region. Although per capita consumption is still quite low in the Mediterranean region, avocado intake has radically increased in the region over the last 5 years. Between 2013 and 2018, per capita consumption of avocados increased 267% in Spain from 0.3 to 1.1 kg, increased 300% in Italy from 0.1 to 0.4 kg, increased 200% in Greece from 0.2 to 0.6 kg, and increased 50% in France from 1.2 to 1.8 kg (49). Overall, per capita consumption in the European Union increased 150% from 0.4 to 1.0 kg (49). In comparison, per capita consumption in the United States increased 28% over the same time period from 2.5 to 3.2 kg (50).

Finding easy and familiar ways to incorporate avocado into traditional Mediterranean dishes may ease the adoption of the fruit. Incorporating avocados into the Mediterranean diet may also make the dietary pattern more adaptable to populations outside of the Mediterranean region, including those more familiar with consuming avocados.

## Conclusions

Avocados are a healthy and nutritious fruit which are well-suited for growth in the Mediterranean region. Consumption is increasing across the Mediterranean region and eating avocados may help people preserve a nutrient profile that is similar to the traditional Mediterranean dietary pattern. Additionally, incorporation of non-traditional fruits such as the avocado may make the dietary pattern more adaptable to populations outside of the Mediterranean region and increase ease of adherence by incorporating fruits that are already familiar. Research is needed to address these hypotheses. Lastly, adding avocados to clinical food assessments and diet scores may improve disease/mortality predictions at the population level.

## Data Availability Statement

The original contributions presented in the study are included in the article/supplementary material, further inquiries can be directed to the corresponding author.

## Author Contributions

NF conceived of the work, contributed to the writing, and offered critical comments. AL wrote the first draft of the manuscript and provided critital revisions to the content. All authors read and approved the final manuscript.

## Conflict of Interest

NF is an employee of the Hass Avocado Board. AL received payment from the Hass Avocado board for writing and editorial services.

## References

[1] Trichopoulou A Lagiou P. Healthy traditional mediterranean diet: an expression of culture, history, and lifestyle. Nutr Rev. (1997) 55:383–9. 10.1111/j.1753-4887.1997.tb01578.x 9420448

[2] Hoffman R Gerber M. Evaluating and adapting the mediterranean diet for non-Mediterranean populations: a critical appraisal. Nutr Rev. (2013) 71:573–84. 10.1111/nure.12040 24032362

[3] Oldways. Oldways Mediterranean Diet Pyramid. Available online at: https://oldwayspt.org/resources/oldways-mediterranean-diet-pyramid (accessed January 13, 2020).

[4] Castro-Quezada I Roman-Vinas B Serra-Majem L. The mediterranean diet and nutritional adequacy: a review. Nutrients. (2014) 6:231–48. 10.3390/nu6010231 24394536 PMC3916858

[5] da Silva R Bach-Faig A Raido Quintana B Buckland G Vaz de Almeida MD Serra-Majem L. Worldwide variation of adherence to the mediterranean diet, in 1961-1965 and 2000-2003. Public Health Nutr. (2009) 12:1676–84. 10.1017/S1368980009990541 19689839

[6] Leon-Munoz LM Guallar-Castillon P Graciani A Lopez-Garcia E Mesas AE Aguilera MT. Adherence to the mediterranean diet pattern has declined in Spanish adults. J Nutr. (2012) 142:1843–50. 10.3945/jn.112.164616 22875552

[7] Martinez-Gonzalez MA Gea A Ruiz-Canela M. The mediterranean diet and cardiovascular health. Circ Res. (2019) 124:779–98. 10.1161/CIRCRESAHA.118.313348 30817261

[8] Sofi F Cesari F Abbate R Gensini GF Casini A Adherence to mediterranean diet and health status: meta-analysis. BMJ. (2008) 337:a1344 10.1136/bmj.a1344 18786971 PMC2533524

[9] Sofi F Macchi C Abbate R Gensini GF Casini A. Mediterranean diet and health status: an updated meta-analysis and a proposal for a literature-based adherence score. Public Health Nutr. (2014) 17:2769–82. 10.1017/S1368980013003169 24476641 PMC10282340

[10] Schwingshackl L Missbach B Konig J Hoffmann G. Adherence to a mediterranean diet and risk of diabetes: a systematic review and meta-analysis. Public Health Nutr. (2015) 18:1292–9. 10.1017/S1368980014001542 25145972 PMC10273006

[11] Koloverou E Esposito K Giugliano D Panagiotakos D. The effect of mediterranean diet on the development of type 2 diabetes mellitus: a meta-analysis of 10 prospective studies and 136,846 participants. Metabolism. (2014) 63:903–11. 10.1016/j.metabol.2014.04.010 24931280

[12] Schwingshackl L Hoffmann G. Adherence to mediterranean diet and risk of cancer: an updated systematic review and meta-analysis of observational studies. Cancer Med. (2015) 4:1933–47. 10.1002/cam4.539 26471010 PMC5123783

[13] Psaltopoulou T Sergentanis TN Panagiotakos DB Sergentanis IN Kosti R Scarmeas N. Mediterranean diet, stroke, cognitive impairment, and depression: a meta-analysis. Ann Neurol. (2013) 74:580–91. 10.1002/ana.23944 23720230

[14] Trichopoulou A Martinez-Gonzalez MA Tong TY Forouhi NG Khandelwal S Prabhakaran D. Definitions and potential health benefits of the mediterranean diet: views from experts around the world. BMC Med. (2014) 12:112. 10.1186/1741-7015-12-112 25055810 PMC4222885

[15] World Health Organization Healthy Diet 2018. (2018). Available online at: https://www.who.int/en/news-room/fact-sheets/detail/healthy-diet (accessed January 13, 2020).

[16] Avocados raw, California [FoodData Central] [Internet]. (2019). Available online at: fdc.nal.usda.gov (accessed January 13, 2020).

[17] Bhuyan DJ Alsherbiny MA Perera S Low M Basu A Devi OA. The odyssey of bioactive compounds in avocado (Persea americana) and their health benefits. Antioxidants. (2019) 8:426. 10.3390/antiox8100426 31554332 PMC6826385

[18] Lee EA Angka L Rota SG Hanlon T Mitchell A Hurren R. Targeting mitochondria with avocatin B induces selective leukemia cell death. Cancer Res. (2015) 75:2478–88. 10.1158/0008-5472.CAN-14-2676 26077472

[19] Dreher ML. Whole fruits and fruit fiber emerging health effects. Nutrients. (2018) 10:1833. 10.3390/nu10121833 30487459 PMC6315720

[20] Ahmed N Tcheng M Roma A Buraczynski M Jayanth P Rea K. Avocatin B protects against lipotoxicity and improves insulin sensitivity in diet-induced obesity. Mol Nutr Food Res. 2019:e1900688. 10.1002/mnfr.201900688 31609072

[21] Ameer K. Avocado as a major dietary source of antioxidants and its preventive role in neurodegenerative diseases. Adv Neurobiol. (2016) 12:337–54. 10.1007/978-3-319-28383-8_18 27651262

[22] Aune D Giovannucci E Boffetta P Fadnes LT Keum N Norat T. Fruit and vegetable intake and the risk of cardiovascular disease, total cancer and all-cause mortality-a systematic review and dose-response meta-analysis of prospective studies. Int J Epidemiol. (2017) 46:1029–56. 10.1093/ije/dyw319 28338764 PMC5837313

[23] Schwingshackl L Hoffmann G Kalle-Uhlmann T Arregui M Buijsse B Boeing H. Fruit and vegetable consumption and changes in anthropometric variables in adult populations: a systematic review and meta-analysis of prospective cohort studies. PLoS ONE. (2015) 10:e0140846. 10.1371/journal.pone.0140846 26474158 PMC4608571

[24] Hartley L Igbinedion E Holmes J Flowers N Thorogood M Clarke A. Increased consumption of fruit and vegetables for the primary prevention of cardiovascular diseases. Cochrane Database Syst Rev. (2013) 2013:CD009874. 10.1002/14651858.CD009874.pub2 23736950 PMC6464871

[25] FAO. Fats and fatty acids in human nutrition. Report of an expert consultation. FAO Food Nutr Pap. (2010). 91:1–166. 21812367

[26] Arnett DK Blumenthal RS Albert MA Buroker AB Goldberger ZD Hahn EJ ACC/AHA guideline on the primary prevention of cardiovascular disease: a report of the American college of cardiology/American heart association task force on clinical practice guidelines. Circulation. (2019). 140:e596–646. 10.1161/CIR.0000000000000725 30879355 PMC7734661

[27] U.S. Department of Health and Human Services and U.S. Department of Agriculture. 2015–2020 Dietary Guidelines for Americans. U.S. Department of Health and Human Services and U.S. Department of Agriculture. (2015) Available online at: http://health.gov/dietaryguidelines/2015/guidelines/ (accessed January 13, 2020).

[28] Wang DD Li Y Chiuve SE Stampfer MJ Manson JE Rimm EB. Association of specific dietary fats with total and cause-specific mortality. JAMA Int Med. (2016) 176:1134–45. 10.1001/jamainternmed.2016.2417 27379574 PMC5123772

[29] Dreher ML Davenport AJ. Hass avocado composition and potential health effects. Crit Rev Food Sci Nutr. (2013) 53:738–50. 10.1080/10408398.2011.556759 23638933 PMC3664913

[30] Fulgoni VL 3rd Dreher M Davenport AJ. Avocado consumption is associated with better diet quality and nutrient intake, and lower metabolic syndrome risk in US adults: results from the National Health and Nutrition Examination Survey (NHANES) 2001–2008. Nutr J. (2013) 12:1. 10.1186/1475-2891-12-1 23282226 PMC3545982

[31] Unlu NZ Bohn T Clinton SK Schwartz SJ Carotenoid absorption from salad and salsa by humans is enhanced by the addition of avocado or avocado oil. J Nutr. (2005) 135:431–6. 10.1093/jn/135.3.431 15735074

[32] Kopec RE Cooperstone JL Schweiggert RM Young GS Harrison EH Francis DM. Avocado consumption enhances human postprandial provitamin A absorption and conversion from a novel high-beta-carotene tomato sauce and from carrots. J Nutr. (2014) 144:1158–66. 10.3945/jn.113.187674 24899156 PMC4093981

[33] Johnson EJ. Role of lutein and zeaxanthin in visual and cognitive function throughout the lifespan. Nutr Rev. (2014) 72:605–12. 10.1111/nure.12133 25109868

[34] Scott TM Rasmussen HM Chen O Johnson EJ. Avocado consumption increases macular pigment density in older adults: a randomized, controlled trial. Nutrients. (2017) 9:919. 10.3390/nu9090919 28832514 PMC5622679

[35] Johnson EJ Chung HY Caldarella SM Snodderly DM. The influence of supplemental lutein and docosahexaenoic acid on serum, lipoproteins, and macular pigmentation. Am J Clin Nutr. (2008) 87:1521–9. 10.1093/ajcn/87.5.1521 18469279

[36] Wang L Bordi PL Fleming JA Hill AM Kris-Etherton PM. Effect of a moderate fat diet with and without avocados on lipoprotein particle number, size and subclasses in overweight and obese adults: a randomized, controlled trial. J Am Heart Assoc. (2015) 4:e001355. 10.1161/JAHA.114.001355 25567051 PMC4330060

[37] Mahmassani HA Avendano EE Raman G Johnson EJ. Avocado consumption and risk factors for heart disease: a systematic review and meta-analysis. Am J Clin Nutr. (2018) 107:523–36. 10.1093/ajcn/nqx078 29635493

[38] Peou S Milliard-Hasting B Shah SA. Impact of avocado-enriched diets on plasma lipoproteins: a meta-analysis. J Clin Lipidol. (2016) 10:161–71. 10.1016/j.jacl.2015.10.011 26892133

[39] Wang L Tao L Hao L Stanley TH Huang KH Lambert JD. A moderate-fat diet with one avocado per day increases plasma antioxidants and decreases the oxidation of small, dense LDL in adults with overweight and obesity: a randomized controlled trial. J Nutr. (2019) 150:276–84. 10.1093/jn/nxz231 31616932 PMC7373821

[40] Park E Edirisinghe I Burton-Freeman B. Avocado fruit on postprandial markers of cardio-metabolic risk: a randomized controlled dose response trial in overweight and obese men and women. Nutrients. (2018) 10:1287. 10.3390/nu10091287 30213052 PMC6164649

[41] Wien M Haddad E Oda K Sabate J. A randomized 3x3 crossover study to evaluate the effect of Hass avocado intake on post-ingestive satiety, glucose and insulin levels, and subsequent energy intake in overweight adults. Nutr J. (2013) 12:155. 10.1186/1475-2891-12-155 24279738 PMC4222592

[42] O'Neil CE Nicklas TA Fulgoni VL 3rd Avocado consumption by adults is associated with better nutrient intake, diet quality, and some measures of adiposity: National health and nutrition examination survey, 2001-2012. Int Med Rev. (2017). 3:422 10.18103/imr.v3i4.422

[43] Heskey C Oda K Sabate J. Avocado intake, and longitudinal weight and body mass index changes in an adult cohort. Nutrients. (2019) 11:691. 10.3390/nu11030691 30909592 PMC6471050

[44] Henning SM Yang J Woo SL Lee RP Huang J Rasmusen A. Hass avocado inclusion in a weight-loss diet supported weight loss and altered gut microbiota: a 12-week randomized, parallel-controlled trial. Curr Dev Nutr. (2019) 3:nzz068. 10.1093/cdn/nzz068 31367691 PMC6658913

[45] Zhu L Huang Y Edirisinghe I Park E Burton-Freeman B. Using the avocado to test the satiety effects of a fat-fiber combination in place of carbohydrate energy in a breakfast meal in overweight and obese men and women: a randomized clinical trial. Nutrients. (2019) 11:952. 10.3390/nu11050952 31035472 PMC6567160

[46] Carman HF The story behind avocado's rise to prominence in the United States. ARE Update. (2019) 22:9–11.

[47] FruiTrop CIRAD The French Agricultural Research Center for International Development. FruiTrop. Available online at: https://www.fruitrop.com/enp (accessed January 13, 2020).

[48] Linden T Spain steadily growing volume as it gains access to the US market. Grove. 2014:37–8.

[49] Fruit Logistica Messe Berlin GmbH European Statistics Handbook. Berlin, Germany: Fruit Logistica, Messe Berlin GmbH (2019).

[50] United States Department of Agriculture Economic Research Service Fruit and Tree Nut Yearbook Tables. Available online at: https://www.ers.usda.gov/data-products/fruit-and-tree-nut-data/fruit-and-tree-nut-yearbook-tables/#General (accessed January 13, 2020).

